# Analysis of Bipolar Radiofrequency Ablation in Treatment of Atrial Fibrillation Associated with Rheumatic Heart Disease

**DOI:** 10.1371/journal.pone.0151248

**Published:** 2016-03-09

**Authors:** Xiliang Zhu, Qian Li, Yang Li, Zhong Wu

**Affiliations:** 1 Department of Cardiovascular Surgery, West China Hospital, Sichuan University, Chengdu, Sichuan Province, P. R. China; 2 West China School of Medicine, Sichuan University, Chengdu, Sichuan Province, P. R. China; Shenzhen institutes of advanced technology, CHINA

## Abstract

**Background:**

Among patients with rheumatic heart disease (RHD), 45% to 60% present with atrial fibrillation (AF), which is associated with increased rates of thromboembolism, heart failure, and even death. The bipolar radiofrequency ablation (BRFA) combining with mitral valve procedure has been adopted in patients of AF associated with RHD, but evaluations about its effectiveness are still limited.

**Methods:**

A total of 87 patients with RHD and long persistent AF who had accepted mitral valve replacement concomitant with BRFA were studied. Clinical data were collected to analyze the midterm results of BRFA and evaluate its efficiency. Univariate and multivariate analyses were used to identify the independent factors associated with late AF recurrence.

**Results:**

Sixty-six (75.9%) patients maintained sinus rhythm after a mean follow-up of 13.4 ± 5.2 months. Late AF recurrence had been detected in 21 (24.1%) patients, 11 (12.6%) patients were confirmed to be AF, 8 (9.2%) patients were atrial flutter and 2 (2.3%) patients were junctional rhythm. In Multivariate logistic regression analysis, body mass index (BMI) (*OR* = 1.756, 95% *CI* = 1.289–2.391, *p* = 0.000) and early AF recurrence (*OR* = 5.479, 95% *CI* = 1.189–25.254, *p* = 0.029) were independent predictors of late AF recurrence. In addition, left ventricular ejection fraction (LVEF) and New York Heart Association class showed a greater improvement in patients who maintained sinus rhythm than those who experienced late AF recurrence.

**Conclusion:**

BRFA is an effective technique for the treatment of long persistent AF associated with RHD during mitral valve replacement. The BMI and early AF recurrence are independent predictors for late AF recurrence. Patients with long-term restoration of sinus rhythm experienced a greater improvement of left ventricular function after BRFA.

## 1. Introduction

Atrial fibrillation (AF) is the most common types of cardiac arrhythmia, which is characterized by chaotic electrical activity, lack of coordinated atrial contractility and an irregular R-R interval [1, 2]. The prevalence of it is estimated to be 0.4% in general population and up to 45%-60% among patients with rheumatic heart disease (RHD) [3, 4]. In the United States, over 3 million people presently suffer from AF, and in Europe the corresponding number is almost 6 million, and the number is estimated to keep growing over the next few years [5, 6]. Patients with AF lose atrial pump function and their Left ventricular systolic function are also impaired, thus reducing cardiac output. AF has been proved to be close association with reduced quality of life, increased rates of thromboembolism, heart failure, and even death [6, 7].

AF is usually caused by cardiac structural abnormalities, atrial electrophysiological abnormalities, or both of them [5]. Currently, it is believed that AF is triggered and maintained as a result of multiple wavelet reentrant circuits, and rotors or spiral wave reentrant circuits in left and right atrium [7]. The traditional “cut-and-sew” maze operation, which was pioneered by Dr Cox, was designed to block these reentrant circuits and fibrillatory conduction [3, 6]. Though it is an effective method for surgical treatment of AF, the application of it is limited due to complex surgical procedures, increased cross-clamp time and high risks of bleeding, sick sinus syndrome, and myocardial dysfunction [2, 3].

The bipolar radiofrequency ablation (BRFA) has been introduced as an attempt to alleviate those problems. It simplifies the traditional maze operation by replacing the complex surgical incisions with lines of transmural necrosis, which can effectively avoid complications of the traditional “cut-and-sew” method [2]. Previous studies have reported that during a median follow-up of twelve months, success rates for restoring sinus rhythm of AF patients with the BRFA operation ranged from 54% to 90% [8, 9]. However, most patients of these studies had degenerative mitral regurgitation, and the data are still scant on the efficacy of BRFA in patients of AF associated with RHD[10–14]. Therefore, the objective of this study is to assess the midterm results of BRFA concomitant with mitral valve replacement (MVR) in treating long persistent AF resulted from RHD and further identify the potential predictors of late AF recurrence.

## 2. Materials and Methods

### 2.1 Patients and Data Collection

From January 2013 to July 2015, a selective group of patients underwent BRFA (Isolators and Glidepath tape; Atricure Inc, Cincinnati, Ohio) and concomitant MVR for treating long persistent AF combined with RHD at West China Hospital of Sichuan University were recruited in this study. Long persistent AF was defined as that AF had been presented for more than 6 months [8, 15–16]. Patients were examined with 12-lead surface electrocardiogram (ECG) and/or 24 hour holter monitoring. The medical histories of patients were carefully examined.

Routine preoperative examinations were performed including transthoracic echocardiography, chest X-ray film. All patients provided blood samples for measurements of thyroid function tests, hepatic and renal functions tests, high sensitive C-reactive protein levels (hs-CRP), N-terminal pro brain natriuretic peptide levels (NT-pro BNP), erythrocyte sedimentation rate (ESR), serum creatinine, *etc*. Included criteria for patients were as follows: 1) aged at 18 years old or above; 2) AF lasted over 6 months; 3) with rheumatic heart disease; 4) received BRFA using Atricure apparatus; and 5) concomitant with MVR. Patients were excluded if they: 1) had AF duration less than 6 months; 2) aged over 80 or less than 18 years old; 3) had mitral regurgitation for the reason of hypertrophic cardiomyopathy or ischemic heart diseases; 4) implanted with permanent pacemaker after BRFA; and 5) used unipolar radiofrequency ablation. Ultimately, eighty-seven consecutive patients were eligible for this study.

This retrospective study followed the tenets of Declaration of Helsinki and was approved by the ethics review board of West China Hospital, Sichuan University. The written informed consent was obtained from all patients at the time of admission, and the information of patients was anonymized and de-identified prior to analysis.

### 2.2 Surgical approaches

**Fig 1:**
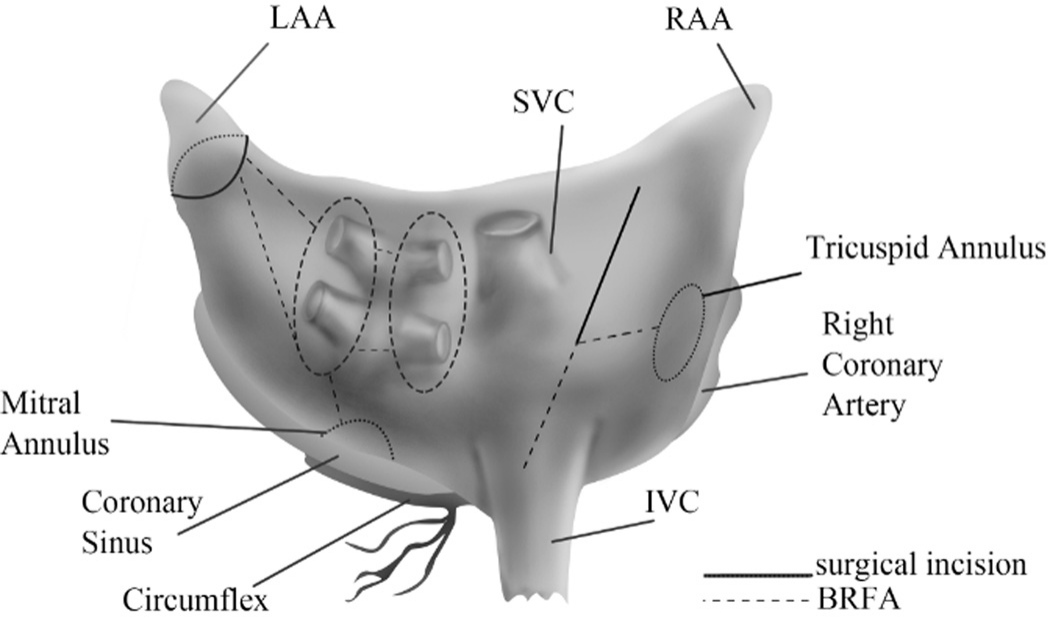
Schematic representation of lesion sets created with BRFA. BRFA: bipolar radiofrequency ablation; LAA: left atrial appendage; RAA: right atrial appendage; IVC: inferior vena cava; SVC: superior vena cava.

The operations were performed through median sternotomy, with cardiopulmonary bypass. Cold blood cardioplegia solution was perfused through the aortic root and the coronary sinus for myocardial protection. MVR was performed by the way of right atrium and interatrial septum. Aortic valve replacement (AVR) was undergone via the incision of ascending aorta. MVR and AVR were performed in interrupted suture. Tricuspid valve repair was conducted for patients with tricuspid regurgitation. For BRFA, the Atricure BRFA apparatus was used according to the procedures described by Sims et al [17]. Radiofrequency lesions were created in an epicardial manner and the ablation lines were described in Fig 1 [18]. In order to block the reentry loops in the left atrium, additional ablation lines were performed between left atrial appendage and left inferior pulmonary veins, and between left inferior pulmonary veins and the posterior leaflets of the mitral valve. Temporary pacemakers were placed for all patients and were used when heart rate was less than 70 beats per minute.

### 2.3 Postoperative management and follow-up

After the procedure, anti-arrhythmic drug of amiodarone was given to patients on a routine basis. Different patients received this drug at a dose ranging from 720 mg to 1200 mg per day for 2 days by intravenous infusion. After they were transferred out of the intensive care unit, the drug was taken orally at 400 mg per day for one week and then reduced to 200 mg per day for 3 to 6 months except those patients whose heart rate was less than 70 beats per minute. For patients still in AF after BRFA and treatment with amiodarone have no effect, direct current cardioversion was tried during their hospitalization. Warfarin was administered for all patients with an international normalized ratio range from 1.5 to 2.5. It was given for three months for patients with bioprosthesis valve and no AF was detected. Patients with mechanical valves needed to take it for a lifetime no matter of heart rhythm. All patients were followed up by ECG or 24-hour holter monitoring before leaving hospital and at 1, 3, 6 and 12 months after operation in clinic of our hospital. The echocardiography was also evaluated before discharge and at 3 and 12 months after operation. The left atrium diameter (LAD), left ventricle end diastolic diameter (LVEDD), and left ventricular ejection fraction (LVEF) were also collected. We collect these medical data through the electronic medical records.

### 2.4 Statistical Analysis

After evaluating the distribution of continuous variables with Kolmogorov-Smirnov test, normal distributed data were expressed in form of means ± standard deviation (SD) and were analyzed with the Student *t*-test, and other un-normal distributed data were reported with median as well as *Q1*, *Q3*, and were analyzed with the Mann-Whitney *U* test. Categorical data were reported in forms of ratio and were analyzed with the Chi-square. Univariate analyses of relevant risk factors for late AF recurrence were conducted by Chi-square or Fisher's exact tests of categorical data and Student's *t* tests of continuous data to compare the differences between patients with late AF recurrence and those without. Variables with *p* < 0.10 in univariate analyses were incorporated into multivariate logistic regression models to indentify independent predictors of late AF recurrence. A two-sided *p* < 0.05 was regarded as statistically significant. All data were analyzed by Statistic Package for Social Science (SPSS V17.0, Chicago, Illinois, USA).

## 3. Results

### 3.1 Patients characteristics and outcome of BRFA

The characteristics of the 87 patients are shown in Table 1. The mean age of patients was 52.9 ± 9.4 years (range, 31–79 years), 57 (65.5%) cases were female and 20 (34.5%) were male. Among these patients, 26 (29.9%) cases were in New York Heart Association (NYHA) class II and 61 (70.1%) in class III, and the mean AF duration was 4.6 ± 3.9 years (range, 0.6 to 20 years). The follow-up time was 13.4 ± 5.2 months. Mean body mass index (BMI) was 21.9 ± 2.6 (kg/m^2^), and 14 (6.2%) patients had BMI greater than 25 kg/m^2^. Hypertension and diabetes mellitus were present in 19 (21.8%), and 12 (13.8%) patients, respectively. Twenty-six (29.9%) patients had a history of smoking. The mean LAD, LVEDD and LVEF by echocardiography were 48.5 ± 6.6 mm, 47.1 ± 4.9 mm, and 59.6% ± 6.6%, respectively. Only 17 (19.5%) patients had LAD greater than 55 mm, and none of patients had LAD greater than 60 mm. Nineteen (21.8%) of patients' LVEF was less than 55%, and none of patients were less than 40%. The mean hs-CRP and ESR were 3.5 ± 2.6 mg/L and 19.0 ± 15.3 mm/h, respectively. The mean NT-pro BNP was 1423.5 ± 864.5 pg/ml, and only 18 (20.7%) patients had NT-pro BNP more than 2000 pg/mL.

**Table 1:** Clinical characteristics of patients.

Characteristics	Value
Age (years)	52.9 ± 9.4
Female, n (%)	57 (65.5%)
Body mass index (kg/m2)	21.9 ± 2.6
Duration of AF (years)	4.6 ± 3.9
Diabetes mellitus, n (%)	12 (13.8%)
Hypertension, n (%)	19 (21.8%)
Smoking, n (%)	26 (29.9%)
NYHA > II, n (%)	61 (70.1%)
Echocardiography	
LAD (mm)	48.5 ± 6.6
LVEDD (mm)	47.1 ± 4.9
LVEF (%)	59.6 ± 6.6
Laboratory parameters	
ESR (mm/h)	19.0 ± 15.3
Hemoglobin (g/dL)	138.8 ± 15.6
hs-CRP (mg/L)	3.5 ± 2.6
WBC (10^9^/L)	6.5 ± 1.7
NT-pro BNP (pg/mL)	1423.5 ± 864.5
Serum uric acid (umol/L)	404.9 ± 97.9
Medical therapy	
Amiodarones, n (%)	75 (86.2%)
Amiodarones/ß-blockers, n (%)	13 (14.9%)
Early AF recurrence, n (%)	26 (29.9%)
Follow up times (months)	13.4 ± 5.2

AF: atrial fibrillation; LAD: left atrial diameter; LVEDD: left ventricle end diastolic diameter; LVEF: left ventricular ejection fraction; hs-CRP: C-reactive protein levels; NT-pro BNP: N-terminal pro brain natriuretic peptide levels; ESR: erythrocyte sedimentation rate; NYHA: New York Heart Association; WBC: White blood cell count.

According to medical treatment regimens after ablation, 75 (86.2%) patients used amiodarone for 3 month, and 13 patients were treated with β-receptor blocker at the same time. Only 12 (13.8%) patients did not receive any antiarrhythmic medicine for bradycardia. Early AF recurrence occurred in 26 (29.9%) patients in the first month of follow-up, whereas 11 (42.3%) patients had been identified as delayed cure in subsequent follow-up time. Late AF recurrence had been detected in 21 (24.1%) patients, 11 patients were confirmed to be AF, 8 patients were atrial flutter and 2 patients were junctional rhythm. Finally, 66 (75.9%) patients maintained sinus rhythm until the last follow-up.

### 3.2 Univariate analyses of factors associated with late AF recurrence

Differences between patients with late AF recurrence and those without were displayed in Table 2. The results showed that patients with late AF recurrence were more likely to experience early recurrence of AF (*p* = 0.000). The duration of AF history in patients of late AF recurrence was longer than those without late AF recurrence (*p* < 0.10). Patients with late AF recurrence had higher BMI (*p* = 0.000) and LAD (*p* = 0.028), with 10 (47.6%) patients' BMI greater than 25 kg/m^2^ and 6 (28.6%) patients' LAD larger than 55 mm. Patients with late AF recurrence also tended to have higher levels of NT-pro BNP (*p* < 0.10) and hemoglobin (*p* < 0.10). The LAD, AF duration, BMI, early AF recurrence and levels of NT-pro BNP and hemoglobin were significant predictors of late AF recurrence. Other factors, including age, female gender, follow time, LVEF, LVEDD, use of amiodarone and so on were not significant different between patients that experienced late AF recurrence and those who did not.

**Table 2:** Univariate analysis the relating risk factors for late AF recurrence.

Parameters	No LRAF (*n* = 66)	LRAF (*n* = 21)	p* value
Age (years)	52.1 ± 9.8	55.5 ± 7.6	0.143
Female, n (%)	43 (62.2%)	14 (66.7%)	0.899
Body mass index (kg/m^2^)	21.1 ± 2.0	24.3 ± 2.6	0.000
Duration of AF (months)	4.2 ± 3.4	6.0 ± 5.0	0.065
Diabetes mellitus, n (%)	11 (16.7%)	1 (4.8%)	0.310
Hypertension, n (%)	15 (22.7%)	4 (19.0%)	0.722
Smoking, n (%)	20 (30.3%)	6 (28.6%)	0.880
LAD (mm)	47.6 ± 6.8	51.2 ± 5.3	0.028
LVEDD (mm)	47.3 ± 4.7	46.8 ± 5.6	0.678
LVEF (%)	59.2 ± 6.8	60.5 ± 6.3	0.444
ESR (mm/h)	17.7 ± 13.9	23.1 ± 18.8	0.158
Hemoglobin (g/dL)	137.1 ± 16.6	143.0 ± 10.6	0.080
hs-CRP (mg/L)	3.4 ± 2.8	3.9 ± 1.6	0.487
WBC (10^9^/L)	6.4 ± 1.7	6.5 ± 1.9	0.894
NT-pro BNP (pg/mL)	1328.3 ± 909.4	1722.8 ± 633.8	0.068
Serum uric acid (umol/L)	405.3 ± 103.6	403.7 ± 79.7	0.948
Amiodarones, n (%)	57 (86.4%)	18 (85.7%)	1.0
Amiodarones+ß-blockers, n (%)	9 (13.6%)	4 (19.0%)	0.799
Early AF recurrence, n (%)	11 (16.7%)	15 (71.4%)	0.000
Follow up times (years)	13.2 ± 5.2	14.0 ± 5.3	0.515

AF: atrial fibrillation; LRAF: late AF recurrence; LAD: left atrial diameter; LVEDD: left ventricle end diastolic diameter; LVEF: left ventricular ejection fraction; hs-CRP: C-reactive protein levels; NT-pro BNP: N-terminal pro-brain Natriuretic peptide levels; ESR: erythrocyte sedimentation rate; WBC: white blood cell count.

*Independent samples *t* -test for continuous variables and Chi-square test for categorical data.

### 3.3 Multivariate analyses of independent factors associated with late AF recurrence

**Fig 2:**
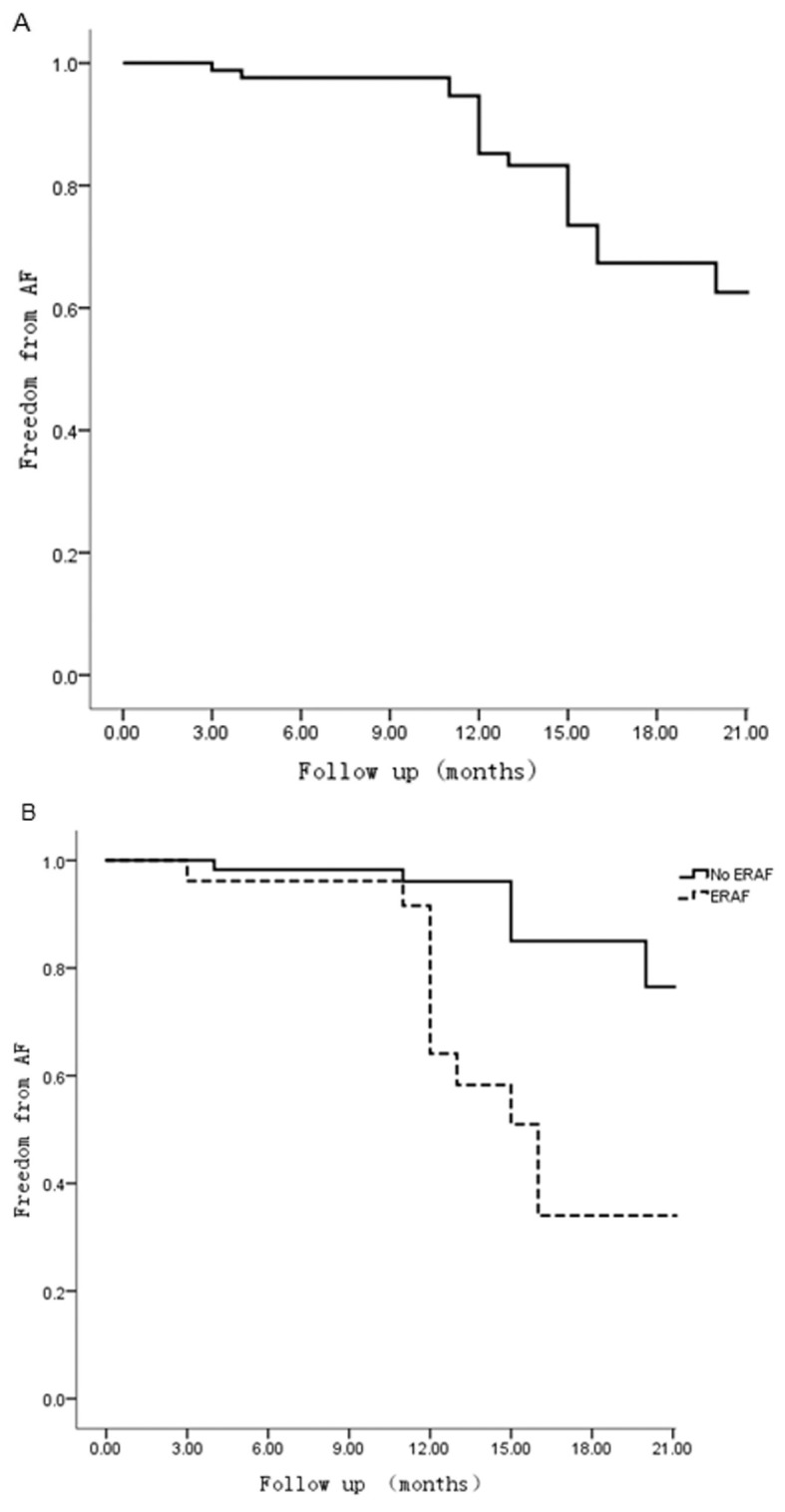
Kaplan-Meier Curves estimates of patients freedom from AF after BRAF. (A) Freedom from AF after BRFA in overall patients. (B) Freedom from AF after BRAF in patients with and without late AF recurrence (Log rank *p* test = 0.000). AF: atrial fibrillation; BRFA: bipolar radiofrequency ablation; LRAD: late recurrence of AF.

Multivariate analysis of the risk factors for late AF recurrence was shown in Table 3. In the univariate analysis, the LAD, AF duration, BMI, early AF recurrence and levels of NT-pro BNP and hemoglobin were significant predictors of late AF recurrence. Those factors were put into multivariate logistic regression models as independent variables. The results showed that BMI (*OR* = 1.756, 95% *CI* = 1.289–2.391, *p* = 0.000) and early AF recurrence (*OR* = 5.479, 95% *CI* = 1.189–25.254, *p* = 0.029) were independent predictors of late AF recurrence. The Kaplan–Meier curve estimates of overall rate free from AF after BRAF is displayed in Fig 2A. For patients with and without early AF recurrence, rates of free from AF are shown in Fig 2B. Patients who suffered from early AF recurrence were at a higher risk of late AF recurrence (log-rank *p* test = 0.000).

**Table 3:** Multivariate analysis the relating risk factors for late AF recurrence.

Parameters	*p* value	*OR*	95% *CI*
Body mass index (kg/m^2^)	0.000	1.756	1.289–2.391
Duration of AF (months)	0.277	1.110	0.919–1.342
LAD (mm)	0.154	1.101	0.965–1.257
Hemoglobin (g/dL)	0.123	1.041	0.989–1.097
NT-pro BNP (pg/mL)	0.082	1.001	1.000–1.002
Early AF recurrence, n (%)	0.029	5.479	1.189–25.254

AF: atrial fibrillation; LAD: left atrial diameter; NT-pro BNP: N-terminal pro brain natriuretic peptide levels.

### 3.4 Clinical impact

**Fig 3:**
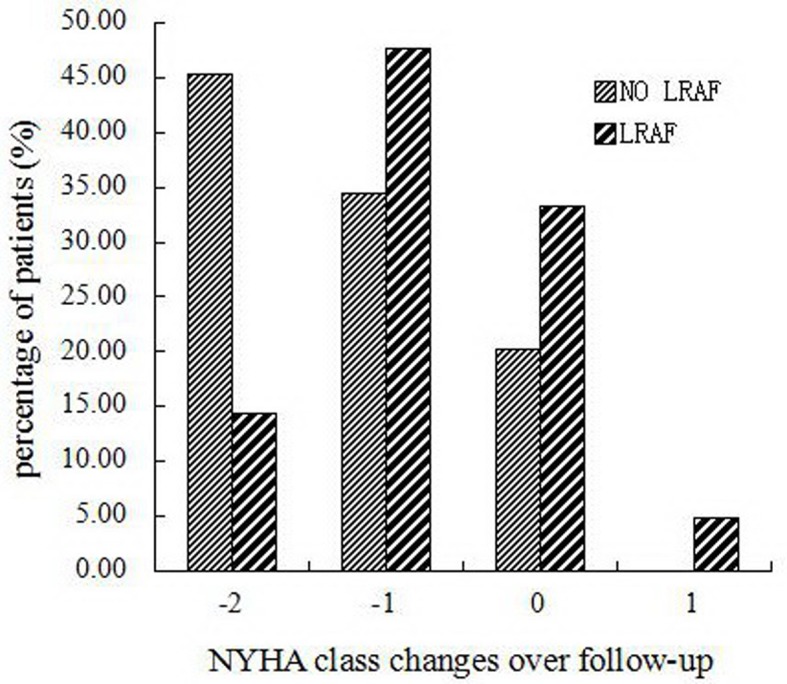
Comparison of NYHA class changes in patients with and without LRAF. Decrease in NYHA class after BRFA were significantly greater in patient without LRAF (*p* = 0.006). BRFA: bipolar radiofrequency ablation; LRAF: late recurrence of AF; NYHA: New York Heart Association.

Changes in echocardiographic parameters and NYHA class between preoperation and postoperation were shown in Table 4. Compared with preoperation, LAD (*p* = 0.002) significantly decreased after AF ablation in the overall population, and a significantly improvement in LVEF (*p* = 0.000) and NYHA class (*p* = 0.000) were also observed during follow-up. In addition, the LVEF showed a greater improvement in patients who maintained sinus rhythm than those who experienced late recurrence of AF (8.4% ± 8.2% VS 3.6 ± 10.3%, *p* = 0.029). Patients without late AF recurrence also found a greater improvement of NYHA class (*p* = 0.006, Fig 3). However, changes in LAD and LVEDD did not show any significant differences.

**Table 4:** Changes in echocardiographic parameters and NYHA class between preoperation and late postoperatively.

Parameters	preoperation	postoperatively	p* value
LAD (mm)	48.5 ± 6.6	46.5 ± 5.1	0.002
LVEDD (mm)	47.1 ± 4.9	46.2 ± 2.4	0.132
LVEF (%)	59.6 ± 6.6	66.8 ± 5.9	0.000
NYHA class			0.000
I	0	49(56.3%)	
II	26 (29.9%)	26 (29.9%)	
III	61 (70.1%)	12(13.8%)	

LAD: left atrial diameter; LVEF: left ventricular ejection fraction; LVEDD: left ventricle end diastolic diameter; NYHA: New York Heart Association.

*Independent samples *t*-test for continuous variables and Chisquare test for categorical data.

## 4. Discussion

In this study, we evaluated the midterm results of BRFA combined with MVR in treating long persistent AF associated with RHD and further identified the predictors of late AF recurrence. Our results showed that after a mean follow-up of 13.4 ± 5.2 months, 75.9% of patients maintained sinus rhythm at time of last follow up. BMI and early AF recurrence were independent predictors for late AF recurrence. Furthermore, patients who maintained sinus rhythm at last time of follow-up showed greater improvement in LVEF and NYHA class than those who experienced late AF recurrence.

AF is a common complication of RHD and the risk of it increases along with the severity of mitral valve lesion [19–20]. Previous studies indicated that RHD not only affected the heart valves, but also caused chronic inflammation and platelet activation, leading to fibrosis and muscle fibers in the atria [19]. Electrophysiological and electroanatomical abnormalities within the left atrium have been detected in these patients, resulting into a more inducibility of AF [20]. Fukada *et al*. [21] reported that the restoration of sinus rhythm was less satisfactory in patients with RHD than patients with non-RHD, and they suggested that the indications for the ablation of AF resulted from rheumatic mitral stenosis should be reconsidered. Similarly, another study with a mean follow-up time of 7.1 ± 2.8 years, only 47% of patients were in sinus rhythm. The authors concluded that close follow-up with antiarrhythmic drugs and/or cardioversion was required for the treatment of AF associated with RHD to enhance the success rate [22]. Furthermore, other studies directly indicated that RHD were independent predictors for late AF recurrence [23, 24].

However, several recent studies showed that the BRFA operation provided favorable results in patients with AF combined with RHD during the mitral valve surgery [25–26]. In the procedures of BRFA, a clamp of the device placed with the negative and positive electrodes on either side of the targeted tissues to precisely focus the energy [25]. It produces transmural lesions and leads to electrical isolation in atrial tissue. In a retrospective comparison of ablation using the BRFA in rheumatic versus nonrheumatic patients, the restoration of sinus rhythm was achieved in 67% for rheumatic patients and 70% for nonrheumatic patients [26]. The efficacy of AF ablation was similar in rheumatic and nonrheumatic patients [26, 27]. Dong *et al*. [25] reported that sinus rhythm was maintained in 78.1% of patients maintained sinus rhythm after at least one year of follow-up. In our study, 75.9% of patients maintained sinus rhythm. Our results were consistent with those previous studies. There are no definite explanations for why the restoration of sinus rhythm rate after ablation was different. Baek *et al*. [23] speculated that it may due to the discrepancy of pre and post operative characteristics, such as the duration of AF, LAD, BMI, early AF recurrence and so on. Other researchers also discussed that it may result from advances in ablation techniques and surgical strategies [26, 28–29].

Recent studies have demonstrated that BMI is associated with the occurrence and development of AF [30–32]. Our study found that BMI is an independent predictor for late AF recurrence. Chilukuri *et al*. [31] observed that only 15% of patients with late AF recurrence had normal BMI (< 25 kg/m^2^), and 41% were overweight (≥ 25 and < 30 kg/m^2^), 44% were obese (≥ 30 kg/m^2^). With the multivariate analysis, only BMI was considered to be an independent predictor of late AF recurrence. The increase of BMI is associated with left atrial dilatation, which may contribute to atrial conduction delay and refractoriness heterogeneities. The abnormality of electroanatomical substrate permits the multiple reentrant circuits and rotors or spiral wave reentrant circuits in the left and right atrium, facilitating the vulnerability to AF [32]. Furthermore, as obese patients are considered under the conditions of chronic inflammatory and oxidative stress state, both of them may also lead to late AF recurrence [33].

Early AF recurrence has also been demonstrated to be an independent risk factor for late AF recurrence [34–35]. Lee *et al*. [34] reported that late AF recurrence was detected in 43% patients with early AF recurrence, and suggested that patients experienced early AF recurrence may had higher risk of late AF recurrence. The main cause of early AF recurrence may be transient stimulatory effect of inflammatory factor after the damage of histopathologic tissue caused by ablation, electrical conduction between the left atrium and pulmonary veins reconnection, and transient imbalance of the autonomic nervous system [32]. Early recurrence of AF interrupts the recovery of atrial remodeling after ablation, thus increasing the risk of late AF recurrence [32]. Patients without experience of early AF recurrence are more likely to be away from late AF recurrence. However, delayed cure during subsequent follow-up can be found in patients with early AF recurrence. In our study, 42.3% of patients with early AF recurrence were found delayed cure, which was concordant with previous studies [28, 32]. Although early AF recurrence is associated with late AF recurrence, the existence of it may not necessarily mean failure of ablation.

AF is regarded to aggravate cardiac dysfunction and decrease the quality of life. In the present study, after BRFA of AF concomitant heart valve surgery, we found that NYHA class and LVEF were significantly improved during follow-up, and LAD was significantly reduced. Tasso *et al*. [36] reported that the echocardiographic outcome after ablation showed significant improvement, especially in patients maintained sinus rhythm. They suggested that it may result from the recovery of atrial contraction and the disappearance of irregular ventricular rhythm. Matto *et al*. [37] analyzed the clinical data of 196 patients with LVEF <50% received ablation, and found that Left ventricular systolic function showed a broader relative increase, and the detrimental effect of AF on cardiac function can be reduced by means of ablation. Interestingly, LVEF and NYHA class showed a significantly higher improvement in patients maintained sinus rhythm than those with late AF recurrence in our study. They also highlight the importance of restoring sinus rhythm in patients of AF. BRFA of AF concomitant heart valve surgery appears to be conductive for the recovery of cardiac function.

## 5. Limitations

There are some limitations in our study. First, it is a retrospective study in a single center, lacking of randomization in selection of patients, therefore, selection bias and the lack of retrospective clinical data may affect results [21]. Second, the results of rhythm in some patients were recorded by 12-lead ECG, and the follow-up time was not long enough that some late AF recurrence may not be detected. This may account for some of the reasons of the higher restoration of sinus rhythm rates in comparison with previous reported studies. Third, some of the patients were comorbid with aortic valve disease or coronary heart disease other than isolated rheumatic mitral valve disease. The potential influence of other operation procedures on the results could not be excluded. Fourth, atrial volume is suggested to be a better indicator for restoring sinus rhythm [38]. However, due to the retrospective study, the data of atrial volume could not be obtained. So we used LAD to reflect the size of left atrial. Fifth, magnetic resonance imaging could be used to assess the different of atrial fibrosis and hemodynamics in these patients, which are independent predictor of arrhythmia recurrences [39–41]. It is not included in this study and will be conducted in the further study.

## 6. Conclusions

BRFA is an effective technique for the treatment of AF resulted from RHD, and long-term restoration of sinus rhythm is associated with greater improvement of LV function. The BMI and early AF recurrence are independent predictors for late recurrence of AF. Future well-designed prospective studies with a large sample size and detailed analyses of potential predictors for late AF recurrence are still warranted to confirm our findings.

## Supporting Information

S1 Checklist PLOSOne_Clinical_Studies_Checklist. (DOCX)

S2 Checklist STROBE_checklist_v4_combined_PlosMedicine. (DOC)
