# Supplementary material for: Analysis of Bipolar Radiofrequency Ablation in Treatment of Atrial Fibrillation Associated with Rheumatic Heart Disease
Source: PLoS One. 2016 Mar 9;11(3):e0151248. doi: 10.1371/journal.pone.0151248 (PMC4784895; doi:10.1371/journal.pone.0151248)
Supplement: S2 Checklist — (DOC) [file pone.0151248.s002.doc]

**S2 Checklist. STROBE_checklist_v4_combined_PlosMedicine**

STROBE Statement—checklist of items that should be included in reports of observational studies

|  | Item No. | Recommendation | Page  No. | Relevant text from manuscript |
| --- | --- | --- | --- | --- |
| **Title and abstract** | 1 | (*a*) Indicate the study’s design with a commonly used term in the title or the abstract | 2 | In this retrospective study, a total of 87 patients with RHD and long standing persistent AF who had accepted mitral valve replacement concomitant with BRFA were studied. |
|  |  | (*b*) Provide in the abstract an informative and balanced summary of what was done and what was found | 2 | Clinical information for a total of 87 patients with RHD and long standing persistent AF who accepted mitral valve replacement concomitant with BRFA for treatment were collected to analyze the midterm results of BRFA and evaluate its efficiency. Univariate and multivariate analyses were used to identify the independent factors associated with late AF recurrence.  BRFA is an effective technique for the treatment of long standing persistent AF associated with RHD during mitral valve replacement. The BMI and early AF recurrence are independent predictors for late AF recurrence. Patients with long-term restoration of sinus rhythm experienced a greater improvement of left ventricular function after BRFA. |
| Introduction | | | |  |
| Background/rationale | 2 | Explain the scientific background and rationale for the investigation being reported | 4-5 | This kind of cardiac arrhythmia is usually caused by cardiac structural abnormalities, atrial electrophysiological abnormalities, or both of them. Currently, it is generally believed that AF is triggered and maintained as a result of multiple wavelet reentrant circuits, and rotors or spiral wave reentrant circuits in the left and right atrium. The traditional “cut-and-sew” maze operation, which was pioneered by Dr Cox, was designed to block these reentrant circuits and fibrillatory conduction. Though it is an effective method for surgical treatment of AF, the application of it is limited due to complex surgical procedures, increased cross-clamp time and high risks of bleeding, sick sinus syndrome, and myocardial dysfunction.  The bipolar radiofrequency ablation (BRFA) has been introduced as an attempt to alleviate those problems. It simplifies the traditional maze operation by replacing the complex surgical incisions with lines of transmural necrosis, which can effectively avoid complications of the traditional “cut-and-sew” method. During a median follow-up of at least twelve months, success rates for restoring sinus rhythm of AF patients with the BRFA operation ranged from 54% to 90%. |
| Objectives | 3 | State specific objectives, including any prespecified hypotheses | 5 | The objective of this study is to assess the midterm results of BRFA concomitant with mitral valve replacement (MVR) in treating long standing persistent AF associated with RHD and further identify the potential predictors of late AF recurrence. |
| Methods | | | |  |
| Study design | 4 | Present key elements of study design early in the paper | 6 | In this retrospective study, a selective group of patients underwent BRFA (Isolators and Glidepath tape; Atricure Inc, Cincinnati, Ohio) and concomitant MVR for treating long standing persistent AF combined with RHD from January 2013 to January 2015 at West China Hospital of Sichuan University were recruited. |
| Setting | 5 | Describe the setting, locations, and relevant dates, including periods of recruitment, exposure, follow-up, and data collection | 5 | From January 2013 to July 2015, a selective group of patients underwent BRFA (Isolators and Glidepath tape; Atricure Inc, Cincinnati, Ohio) and concomitant MVR for treating long standing persistent AF combined with RHD at West China Hospital of Sichuan University were recruited in this study.  All patients accepted the ECG or 24-hour holter monitoring before leaving hospital and during their follow-up at 1, 3, 6 and 12 months after operation in outpatient clinic of our hospital.  We collected these medical data through the electronic medical records. |
| Participants | 6 | (*a*) *Cohort study*—Give the eligibility criteria, and the sources and methods of selection of participants. Describe methods of follow-up  *Case-control study*—Give the eligibility criteria, and the sources and methods of case ascertainment and control selection. Give the rationale for the choice of cases and controls  *Cross-sectional study*—Give the eligibility criteria, and the sources and methods of selection of participants | 6 | Included criteria for patients were as follows: 1) aged at 18 years old or above; 2) AF lasted over 6 months; 3) with rheumatic mitral lesion; 4) received BRFA using Atricure apparatus; and 5) concomitant with MVR. Patients were excluded if they: 1) had AF duration less than 6 months; 2) aged over 80 or less than 18 years old; 3) had mitral regurgitation for the reason of hypertrophic cardiomyopathy or ischemic heart diseases; 4) implanted with permanent pacemaker after BRFA; and 5) used unipolar radiofrequency ablation.  All patients accepted the ECG or 24-hour holter monitoring before leaving hospital and at 1, 3, 6 and 12 months after operation in outpatient clinic of our hospital. |
|  |  | (*b*) *Cohort study*—For matched studies, give matching criteria and number of exposed and unexposed  *Case-control study*—For matched studies, give matching criteria and the number of controls per case |  |  |
| Variables | 7 | Clearly define all outcomes, exposures, predictors, potential confounders, and effect modifiers. Give diagnostic criteria, if applicable | 6 | Diagnostic criteria: Long standing persistent AF was defined as that AF had been presented for more than 6 months. |
| Data sources/ measurement | 8* | For each variable of interest, give sources of data and details of methods of assessment (measurement). Describe comparability of assessment methods if there is more than one group | *6* | All patients provided blood samples for measurements of thyroid function tests, hepatic and renal functions tests, high sensitive C-reactive protein levels (hs-CRP), N-terminal pro brain natriuretic peptide levels (NT-pro BNP), erythrocyte sedimentation rate (ESR), serum creatinine, etc.  The echocardiography was also evaluated before discharge and at 3 and 12 months after operation. The left atrium diameter (LAD), left ventricle end diastolic diameter (LVEDD), and left ventricular ejection fraction (LVEF) were also collected. |
| Bias | 9 | Describe any efforts to address potential sources of bias | 6 | Patients were excluded if they: 1) had AF duration less than 6 months; 2) aged over 80 or less than 18 years old; 3) had mitral regurgitation for the reason of hypertrophic cardiomyopathy or ischemic heart diseases; 4) implanted with permanent pacemaker after BRFA; and 5) used unipolar radiofrequency ablation. |
| Study size | 10 | Explain how the study size was arrived at | 6 | Although 98 patients met the including criteria, 11 of them were found with irregular out-patient review and their data were excluded. Ultimately, eighty-seven consecutive patients were eligible for this study. |

Continued on next page

| Quantitative variables | 11 | Explain how quantitative variables were handled in the analyses. If applicable, describe which groupings were chosen and why | 8-9 | For measurement data, after evaluating the distribution of continuous variables with Kolmogorov-Smirnov test, normal distributed data were expressed in the form of means ± standard deviation ( SD ) and were analyzed with the Student t-test, and other un-normal distributed data were described with median as well as Q1, Q3, and were analyzed with the Mann-Whitney U test. |
| --- | --- | --- | --- | --- |
| Statistical methods | 12 | (*a*) Describe all statistical methods, including those used to control for confounding | 8-9 | For measurement data, after evaluating the distribution of continuous variables with Kolmogorov-Smirnov test, normal distributed data were expressed in the form of means ± standard deviation (SD) and were analyzed with the Student t-test, and other un-normal distributed data were described with median as well as Q1, Q3, and were analyzed with the Mann-Whitney U test. Categorical data were reported in forms of ratio and were analyzed with the Chi-square. Univariate analyses of relevant risk factors for late AF recurrence were conducted by Chi-square or Fisher's exact tests of categorical data and Student's t tests of continuous data to compare the differences between patients with late AF recurrence and those without. Variables with p < 0.10 in univariate analyses were incorporated into multivariate logistic regression models to indentify independent predictors of late AF recurrence. A two-sided p < 0.05 was regarded as statistically significant. All data were analyzed by Statistic Package for Social Science (SPSS V17.0, Chicago, Illinois, USA). |
|  |  | (*b*) Describe any methods used to examine subgroups and interactions |  | There is not any subgroup in our study. |
|  |  | (*c*) Explain how missing data were addressed | 6 | Although 98 patients met the including criteria, 11 of them were found with irregular out-patient review and their data were excluded. |
|  |  | (*d*) *Cohort study*—If applicable, explain how loss to follow-up was addressed  *Case-control study*—If applicable, explain how matching of cases and controls was addressed  *Cross-sectional study*—If applicable, describe analytical methods taking account of sampling strategy | 6 | Although 98 patients met the including criteria, 11 of them were found with irregular out-patient review and their data were excluded. |
|  |  | (*e*) Describe any sensitivity analyses |  |  |
| Results | | | | |
| Participants | 13* | (a) Report numbers of individuals at each stage of study—eg numbers potentially eligible, examined for eligibility, confirmed eligible, included in the study, completing follow-up, and analysed |  |  |
|  |  | (b) Give reasons for non-participation at each stage |  |  |
|  |  | (c) Consider use of a flow diagram |  |  |
| Descriptive data | 14* | (a) Give characteristics of study participants (eg demographic, clinical, social) and information on exposures and potential confounders | 10 | The characteristics of the 87 patients are shown in Table 1. The mean age of the patients was 52.9 ± 9.4 years (range, 31-79 years), 57 (65.5%) cases were female and 20 (34.5%) were male. Among these patients, 26 (29.9%) cases were in New York Heart Association (NYHA) class II and 61 (70.1%) in class III, and the mean AF duration was 4.6 ± 3.9 years (range, 0.6 to 20 years). The follow-up time was 13.4 ± 5.2 months. Mean body mass index (BMI) was 21.9 ± 2.6 (kg/m2), and 14 (6.2%) patients had BMI greater than 25 kg/m2. Hypertension and diabetes mellitus were present in 19 (21.8%), and 12 (13.8%) patients, respectively. Twenty-six (29.9%) patients had a history of smoking. |
|  |  | (b) Indicate number of participants with missing data for each variable of interest |  |  |
|  |  | (c) *Cohort study*—Summarise follow-up time (eg, average and total amount) |  |  |
| Outcome data | 15* | *Cohort study*—Report numbers of outcome events or summary measures over time |  |  |
|  |  | *Case-control study—*Report numbers in each exposure category, or summary measures of exposure |  |  |
|  |  | *Cross-sectional study—*Report numbers of outcome events or summary measures |  |  |
| Main results | 16 | (*a*) Give unadjusted estimates and, if applicable, confounder-adjusted estimates and their precision (eg, 95% confidence interval). Make clear which confounders were adjusted for and why they were included |  |  |
|  |  | (*b*) Report category boundaries when continuous variables were categorized |  |  |
|  |  | (*c*) If relevant, consider translating estimates of relative risk into absolute risk for a meaningful time period |  |  |

Continued on next page

| Other analyses | 17 | Report other analyses done—eg analyses of subgroups and interactions, and sensitivity analyses |  |  |
| --- | --- | --- | --- | --- |
| Discussion | | | | |
| Key results | 18 | Summarise key results with reference to study objectives | 15 | Our results showed that after a mean follow-up of 13.4 ± 5.2 months, 75.9% of patients maintained sinus rhythm until the last follow up. BMI and early AF recurrence were independent predictors for late AF recurrence. Furthermore, patients who maintained sinus rhythm at follow-up showed greater improvement in LVEF and NYHA class than those who experienced late AF recurrence. |
| Limitations | 19 | Discuss limitations of the study, taking into account sources of potential bias or imprecision. Discuss both direction and magnitude of any potential bias | 19 | There are some limitations in our study. First, it is a retrospective study in a single center, lacking of randomization in selection of patients, therefore, selection bias and the lack of retrospective clinical data may affect results. Second, the results of rhythm in some patients were recorded by 12-lead ECG, and the follow-up time was not long enough that some late AF recurrence may not be detected. This may account for some of the reasons of the higher restoration of SR rates in comparison with previous reported studies. Third, some of the patients were comorbid with aortic valve disease or coronary heart disease other than isolated rheumatic mitral valve disease. The potential influence of other operation procedures on the results could not be excluded. Fourth, atrial volume is suggested to be a better indicator for restoring SR [38]. However, due to the retrospective study, the data of atrial volume could not be obtained. So we used LAD to reflect the size of left atrial. Fifth, magnetic resonance imaging could be used to assess the different of atrial fibrosis and hemodynamics in these patients, which are independent predictor of arrhythmia recurrences [39-41]. It is not included in this study and will be conducted in the further study. |
| Interpretation | 20 | Give a cautious overall interpretation of results considering objectives, limitations, multiplicity of analyses, results from similar studies, and other relevant evidence |  |  |
| Generalisability | 21 | Discuss the generalisability (external validity) of the study results |  |  |
| Other information | |  | | |
| Funding | 22 | Give the source of funding and the role of the funders for the present study and, if applicable, for the original study on which the present article is based |  | This study was supported by the National Natural Science Foundation of China (Grant number 81371638). ZW received the funding. |

*Give information separately for cases and controls in case-control studies and, if applicable, for exposed and unexposed groups in cohort and cross-sectional studies.

**Note:** An Explanation and Elaboration article discusses each checklist item and gives methodological background and published examples of transparent reporting. The STROBE checklist is best used in conjunction with this article (freely available on the Web sites of PLoS Medicine at http://www.plosmedicine.org/, Annals of Internal Medicine at http://www.annals.org/, and Epidemiology at http://www.epidem.com/). Information on the STROBE Initiative is available at www.strobe-statement.org.
